# Case Report: Practice of whole-course pharmaceutical care: a case in hypertrophic cardiomyopathy and a review of the pharmacist’s role

**DOI:** 10.3389/fcvm.2025.1512784

**Published:** 2025-07-10

**Authors:** Liu-Cheng Li, Su Zhang, Jia-Bin Sun, Yao-Yao Xu, Kai-Li Mao

**Affiliations:** ^1^ Department of Pharmacy, Sir Run Run Shaw Hospital, Zhejiang University School of Medicine, Hangzhou, Zhejiang, China; ^2^ Department of Pharmacy, Center for Clinical Pharmacy, Cancer Center, Zhejiang Provincial People’s Hospital, Affiliated People’s Hospital, Hangzhou Medical College, Hangzhou, Zhejiang, China; ^3^ Department of Pharmacy, Deqing People’s Hospital, Huzhou, Zhejiang, China; ^4^ Department of Pharmacy, The People’s Hospital of Pingyang Hospital, Wenzhou, Zhejiang, China; ^5^ Department of Pharmacy, The Quzhou Affiliated Hospital of Wenzhou Medical University, Quzhou People’s Hospital, Quzhou, Zhejiang, China

**Keywords:** hypertrophic cardiomyopathy, treatment, diltiazem, metoprolol, pharmaceutical care

## Abstract

Clinical pharmacists supported clinicians in medication planning and health education for a male patient diagnosed with hypertrophic cardiomyopathy (HCM). Therapeutically, diltiazem was replaced with metoprolol to further alleviate symptoms. Whole-blood exon sequencing revealed a pathogenic mutation in the beta-myosin heavy chain gene (MYH7, OMIM #160760), consistent with dominant inheritance. Furthermore, low-intensity exercise and screening of the patient’s children were recommended. In addition, we provided an overview of current therapeutic approaches, emphasizing potential novel agents and non-pharmaceutical interventions for HCM management. This is the first case report to highlight not only updates in HCM treatment but also the pharmacist’s role in long-term HCM management.

## Introduction

Hypertrophic cardiomyopathy (HCM) is the most common inherited cardiomyopathy, characterized by left ventricular hypertrophy and myocardial fibrosis, particularly affecting adolescents and athletes (1–3). A 2022 study reported HCM cases in 125 countries, estimating a worldwide prevalence of 15–20 million individuals, or approximately 0.2%–0.5% of the population (4). Epidemiological data from the United States further suggest that the incidence of HCM is significantly lower in children compared to adults. The estimated prevalence of HCM is approximately 1.2 per million, with an annual incidence of 1.3 per 100,000 individuals (5). In a study by Ma et al., 15.6% of HCM patients died during a median follow-up period of 5.1 years, with the majority of deaths attributed to cardiovascular causes, heart transplantation, or sudden cardiac death (2). HCM not only leads to the loss of social talents and imposes a burden on healthcare systems, but also spreads within families as a hereditary condition. This results in reduced quality of life and ongoing psychological and financial burden on affected families, highlighting the importance of effective HCM management.

The clinical classification of HCM is primarily based on hemodynamics, genetic characteristics, and the site of hypertrophy. Among these, the hemodynamic classification (distinguishing between obstructive or non-obstructive types) is the most widely used in clinical practice, as it helps guide treatment decisions. Genetic mutations are identified in approximately 60% of HCM cases (6, 7). Common symptoms include exertional dyspnea, chest pain (reported in approximately 40% of cases), dizziness, palpitations, syncope (15%–25%), and sudden cardiac death, with 80% of these deaths caused by ventricular fibrillation (6). However, clinical symptoms of HCM are highly variable, with some individuals remaining asymptomatic throughout life, while others may experience sudden death as their first symptom. In addition, HCM diagnosed during childhood or adolescence is associated with a poorer prognoses compared to adult-onset cases, emphasizing the importance of family screening in hereditary HCM (4). To date, there are few drugs that can eradicate the clinical symptoms of HCM. Therefore, current treatment strategies are aimed at symptom control, improving cardiac function, and slowing disease progression. Advancing clinical practice and treatment updates is crucial for optimizing patient outcomes. Clinical pharmacists play an important role in identifying and solving drug-related issues (8), yet their involvement in HCM management remains unexplored.

This study presents the first hospital-based report detailing the role of clinical pharmacists in formulating a medication plan and delivering health education for a male patient with HCM. In addition, we offer an overview of updated therapeutic strategies, highlighting both potential novel agents and non-pharmaceutical interventions in the management of HCM.

## Case presentation

A 39-year-old male patient was hospitalized in March 2023. He was severely obese, with a body mass index (BMI) of 30 kg/m^3^. Over the past 5 years, he had experienced episodes of chest tightness and chest pain, often accompanied by shortness of breath during intense physical activity or emotional stress. In 2022, an electrocardiogram revealed abnormal Q waves in the lower and anterior wall leads. Cardiac ultrasound showed a thickening of the basal interventricular septum, measuring 17 mm, an increase from 13 mm observed in 2021. After treatment with diltiazem, his symptoms did not improve significantly. The patient reported occasional alcohol consumption and smoking, with no other diagnosed conditions aside from HCM.

On the day of admission, ultrasound examination revealed left atrial enlargement, while the thickest part of the basal segment of the interventricular septum was 18 mm, with a 68% cardiac ejection fraction. Whole-blood exon testing showed the mutation and dominant inheritance of the HCM pathogenic gene in the beta-myosin heavy chain gene (MYH7, OMIM #160760). Myocardial biopsy indicated features of HCM, including hypertrophy and disordered myocytes. Therapeutically, diltiazem (30 mg, q.d.) was replaced with metoprolol (23.75 mg, q.d.) to alleviate symptoms.

## Discussion

### Conventional drug treatment strategies and non-pharmaceutical interventions

The 2023 Guideline for Diagnosis and Treatment of Patients with HCM stated that the main clinical symptoms of HCM are labile dyspnea and chest pain (6). The pathological changes are mainly characterized by hypertrophic and disordered myocardium. Cardiac ultrasound indicates that wall thickness in any part of the left ventricle greater than 15 mm at the end-diastole—or greater than 13 mm in patients with a gene mutation—can be used to diagnose hypertrophic myocardium. In addition, electrocardiogram findings may include abnormal Q waves (18%–53%), ST-T changes, and prolonged QT intervals. A peak pressure gradient between the left ventricular outflow tract and the aorta of less than 30 mmHg at rest or after excitation, combined with the presence of a gene mutation, supports the diagnosis of non-obstructive and hereditary HCM.

For non-obstructive HCM, therapeutic goals include controlling the progression of myocardial hypertrophy, reducing left ventricular filling pressure, alleviating clinical symptoms, avoiding arrhythmias, and treating heart failure. For asymptomatic patients without significant hemodynamic changes, the guidelines recommend clinical follow-up. β-blockers or non-dihydropyridine calcium channel blockers (NDHP-CCB) are suggested to improve cardiac function if there are no contraindications (6). For patients with atrial fibrillation, a CHA_2_DS_2_-VASc score is not required for initial anticoagulant therapy, and a direct oral anticoagulant is preferred. β-blockers, amiodarone, and propafenone can be used to maintain sinus rhythm and control heart rate. For patients who progress to obstructive HCM, treatment strategies also include interventional surgery (percutaneous intraventricular septal myocardial ablation, intraventricular septal radiofrequency ablation, and endoventricular septal radiofrequency ablation), surgical treatments (Morrow surgery, mitral valve-oriented obstruction relief within the left ventricular cavity, and apical or right ventricular myocardial resection), and double-chamber pacemaker implantation.

Non-pharmaceutical interventions, such as changes in lifestyle habits and attention to suitable exercise, are also key components of HCM management. With a BMI of 30 kg/m^2^, the patient was classified as severely obese and was advised to lose weight. A reasonable diet and low-intensity aerobic exercise to enhance cardiopulmonary endurance and promote fat burning are important aspects of obesity intervention. These include controlling calorie intake, reducing saturated fat intake (such as animal fats), and limiting salt and sugar consumption. The patient also smoked and drank alcohol. As smoking is a known risk factor for heart disease, he was advised to quit smoking. Alcohol consumption increases heart rate and can aggravate the condition of HCM; therefore, abstinence from alcohol was recommended. Exercise is a key aspect of clinical pharmacists’ patient education. The 2020 ESC Guidelines on Sports Cardiology and Exercise in Patients with Cardiovascular Disease recommended low-, moderate-, and high-intensity exercise for HCM patients at high, medium, and low risk of sudden cardiac death, respectively (6). Low-intensity exercise is suitable for patients with exercise-related HCM symptoms (high risk). According to the ESC guidelines (9) and the patient's symptoms, only low-intensity activities, such as jogging, cycling, brisk walking, bowling, swimming, and hiking, were recommended. However, recent prospective studies suggest that individuals with HCM who engage in vigorous sports do not necessarily face a higher arrhythmic risk than those who are less active (10). Similarly, athletes with HCM who continue to compete have not shown a significantly increased risk, despite the limited number of cases. As guidelines continue to evolve, they increasingly advocate for a personalized approach and shared decision-making for athletes with HCM who wish to return to play, even though discrepancies still exist.

### Optimization of drug therapy and promising precision therapy

**Figure 1:**
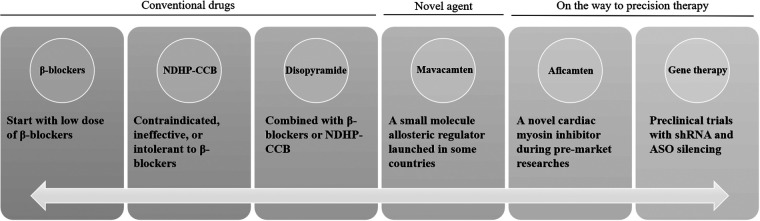
Conventional drugs and promising precision treatment for HCM. ASO, antisense oligonucleotide; HCM, hypertrophic cardiomyopathy; NDHP-CCB, non-dihydropyridine calcium channel blockers; shRNA, short hairpin RNA.

For patients with HCM, rhythm control is considered superior to heart rate control. β-blockers are the first-line treatment in clinical practice (6). First, a low dose of β-blockers should be prescribed based on the patient’s heart rate and blood pressure, provided there are no contraindications to their use (Figure 1). Second, NDHP-CCBs may be considered in patients who are contraindicated, unresponsive, or intolerant to β-blockers. Disopyramide can be used in combination with β-blockers or NDHP-CCBs in patients with significant symptoms. It is recommended that disopyramide be combined with β-blockers or NDHP-CCBs because it may enhance atrioventricular node conduction and increase the ventricular rate. Non-positive inotropic drugs may also be considered when necessary. Vasoconstrictors are mainly used in cases of severe hypotension caused by changes in left ventricular load and can be used in combination with β-blockers. In patients with persistent dyspnea, volume overload, or high left ventricular filling pressure, low-dose oral diuretics are appropriate.

Though researchers have been trying to develop new drugs, the therapeutic agents for HCM are still relatively limited. Mavacamten, a small molecule allosteric regulator recently launched abroad, breaks the dilemma of new drugs in HCM therapy over the past 40 years and marks the beginning of a new era in treatment. The interventional mechanism of mavacamten mainly involves reversible inhibition of actin-myosin cross-bridging, reduction of myocardial contraction, elimination of prephase contraction, and relief of left ventricular outflow tract obstruction (11). A 2023 meta-analysis of randomized controlled trials showed that mavacamten could improve cardiac function in patients with both obstructive and non-obstructive HCM without significant adverse drug reactions (12). However, the largest sample size among those studies was fewer than 300 cases. Since no pharmacokinetic or clinical trials have been conducted in China, it has not yet been approved for marketing there. In a phase 2 study of patients with obstructive HCM, aficamten (a novel cardiac myosin inhibitor) significantly reduced the left ventricular outflow tract gradient and improved N-terminal pro-B-type natriuretic peptide levels (a marker of heart failure) and New York Heart Association functional class in most patients (13). These data highlight the potential of sarcomere-targeted therapy for HCM.

Furthermore, the precision gene therapy strategy is showing valuable results and may offer a promising future for HCM therapy. In human cell models of HCM, short hairpin RNA (shRNA) silencing showed a reduction in the disease's systolic phenotype, as well as disease-associated increases in cardiomyocyte speed, force, and power (14). Antisense oligonucleotide (ASO) silencing was able to target and knock out specific HCM-associated alleles and showed more modest improvements in the contractile phenotype. However, the pathogenic genes of HCM (such as MYH7) may have a complex relationship with various clinical phenotypes, and this needs to be clarified when considering therapeutic allele silencing in relation to different clinical phenotypes. In addition, gene therapy may not be able to reverse HCM and, in the future, early genetic screening of asymptomatic individuals or family members of patients with confirmed genetic mutations (such as parents and children) may lead to early genetic correction and accurate individualized management.

In this research, a young patient with HCM was previously treated with diltiazem, a non-dihydropyridine calcium antagonist, but no significant improvement was observed. After being admitted to our hospital, the patient's heart rate was recorded at 70–81 beats per minute, and there were no other contraindication to metoprolol. Thus, metoprolol (23.75 mg, q.d.) was prescribed to improve ventricular remodeling and control ventricular rate instead of diltiazem, as recommended by the guidelines (6). The dose of metoprolol can be adjusted according to symptoms during later follow-up. If the maximum dose of metoprolol cannot control the symptoms, combination treatment with disopyramide may be considered. It is also important to note that the exon gene sequencing of whole blood showed a mutation and dominant inheritance of the HCM pathogenic gene in the beta-myosin heavy chain gene (MYH7, OMIM #160760). To date, there is still no gene intervention therapy for HCM; however, genetic screening for pathogenic HCM genes in the patient’s children and parents is recommended. This will be conducive to early follow-up and observation, and may help prevent adverse HCM events, such as sudden cardiac death.

Accurate diagnosis of HCM is crucial for its precision therapy. It was reported that the right ventricle may undergo structural and functional adaptations in response to vigorous exercise, known as the athlete's heart, and these changes can resemble those seen in the early stages of arrhythmogenic right ventricular cardiomyopathy (15). Thus, distinguishing early-stage arrhythmogenic right ventricular cardiomyopathy or HCM from exercise-related cardiac adaptations in healthy individuals can be challenging. However, HCM and the athlete’s heart exhibit essential differences in cardiac structure and functional changes. Through clinical manifestations, family history investigations, and the application of various diagnostic tools, the two can be effectively distinguished. Cardiac magnetic resonance imaging findings and techniques can also help differentiate the athlete's heart from HCM (16), the most common cardiomyopathy.

Among other diagnostic tools, an electrocardiogram is an important means of initial screening, as it can reflect abnormal electrical activity of the heart. Echocardiography is a key diagnostic tool that visually assesses the degree of myocardial hypertrophy and changes in cardiac structure and function. For patients suspected of having HCM, ambulatory electrocardiogram monitoring is necessary to closely observe whether arrhythmias occur, which is of great significance for differential diagnosis and disease risk assessment. Cardiac magnetic resonance imaging also holds great value in diagnosing HCM, providing more detailed information on cardiac structure and function – especially for patients with unclear or uncertain echocardiographic results. Therefore, a comprehensive evaluation is essential to identify the pathological characteristics of HCM and assess the risk of sudden cardiac death during physical activity, thereby preventing misdiagnosis. During the treatment process, the results of differential diagnosis should be fully considered to formulate individualized treatment plans and improve patient outcomes and prognosis.

### Risk management of drugs

The 2020 AHA/ACC guidelines suggested that the use of angiotensin-converting enzyme inhibitors (ACEI), angiotensin-II receptor antagonists (ARB), dihydropyridine calcium antagonists, and digoxin may worsen symptoms caused by dynamic efflux tract obstruction in patients with obstructive HCM (17). If the patient develops comorbidities that require the use of these drugs in the future, more caution and enhanced monitoring will be needed. Verapamil is potentially harmful in patients with severe dyspnea at rest, hypotension, and a large pressure gradient (e.g., >100 mmHg) between the left ventricular outflow tract and the aorta at rest (17). The patient in this study was diagnosed with non-obstructive HCM and was taking only metoprolol for symptom control. However, it is necessary to monitor for adverse effects of any additional medications if the disease progresses or becomes complicated by heart failure or other conditions.

## Conclusion

Real-world clinical experience provides valuable insights for the long-term management of HCM (18). In this study, we not only implemented real-world clinical practice and updated HCM treatment strategies but also highlighted the pharmacist’s role in the long-term management of HCM for the first time. Therapeutic options for HCM remain limited. Although new targeted therapeutics such as mavacamten, aficamten, and pathogenic gene silencing approaches offer hope, global data are still lacking, and most evidence comes from preclinical trials. The case presented in this study involved a patient with hereditary HCM. It was recommended that his family members (especially his children) undergo screening for HCM-related pathogenic genes to assess disease risk and help prevent serious outcomes, such as sudden cardiac death. Given the complex clinical presentation and treatment needs of HCM, a multidisciplinary approach is essential. Collaboration among cardiologists, geneticists, nutritionists, rehabilitation therapists, mental health professionals, nurses, and pharmacists is needed. In addition to optimizing drug therapy programs and providing patient education, clinicians and pharmacists should also consider disease progression, stay up to date with drug therapy research, and guide patients using evidence-based non-pharmaceutical interventions to achieve individualized care.

## Data Availability

The original contributions presented in the study are included in the article/Supplementary Material, further inquiries can be directed to the corresponding authors.
